# Bone Marrow Stromal Cells Generate a Pro-Healing Inflammasome When Cultured on Titanium–Aluminum–Vanadium Surfaces with Microscale/Nanoscale Structural Features

**DOI:** 10.3390/biomimetics10010066

**Published:** 2025-01-19

**Authors:** David J. Cohen, Christine M. Van Duyn, Jingyao Deng, Musaddiq K. Lodi, Michelle B. Gallagher, James T. Sugar, Jeremy J. Rawlinson, Preetam Ghosh, Barbara D. Boyan, Zvi Schwartz

**Affiliations:** 1 Department of Biomedical Engineering, Virginia Commonwealth University, Richmond, VA 23284, USA; djcohen@vcu.edu (D.J.C.); cmvanduyn@vcu.edu (C.M.V.D.); dengj3@vcu.edu (J.D.); zschwartz@vcu.edu (Z.S.); 2 Integrative Life Sciences, Virginia Commonwealth University, Richmond, VA 23284, USA; lodimk2@vcu.edu; 3 Medtronic Spine, Memphis, TN 38132, USA; michelle.b.gallagher@medtronic.com (M.B.G.); james.t.sugar@medtronic.com (J.T.S.); jeremy.j.rawlinson@medtronic.com (J.J.R.); 4 Department of Computer Science, Virginia Commonwealth University, Richmond, VA 23284, USA; pghosh@vcu.edu; 5 Georgia Institute of Technology, Atlanta, GA 30332, USA; 6 Department of Periodontics, University of Texas Health Science Center at San Antonio, San Antonio, TX 78229, USA

**Keywords:** biomimetic Ti6Al4V surface topography, RNA-seq, osteogenic media, MSCs, pathway analysis

## Abstract

The surface topography and chemistry of titanium–aluminum–vanadium (Ti6Al4V) implants play critical roles in the osteoblast differentiation of human bone marrow stromal cells (MSCs) and the creation of an osteogenic microenvironment. To assess the effects of a microscale/nanoscale (MN) topography, this study compared the effects of MN-modified, anodized, and smooth Ti6Al4V surfaces on MSC response, and for the first time, directly contrasted MN-induced osteoblast differentiation with culture on tissue culture polystyrene (TCPS) in osteogenic medium (OM). Surface characterization revealed distinct differences in microroughness, composition, and topography among the Ti6Al4V substrates. MSCs on MN surfaces exhibited enhanced osteoblastic differentiation, evidenced by increased expression of RUNX2, SP7, BGLAP, BMP2, and BMPR1A (fold increases: 3.2, 1.8, 1.4, 1.3, and 1.2). The MN surface also induced a pro-healing inflammasome with upregulation of anti-inflammatory mediators (170–200% increase) and downregulation of pro-inflammatory factors (40–82% reduction). Integrin expression shifted towards osteoblast-associated integrins on MN surfaces. RNA-seq analysis revealed distinct gene expression profiles between MSCs on MN surfaces and those in OM, with only 199 shared genes out of over 1000 differentially expressed genes. Pathway analysis showed that MN surfaces promoted bone formation, maturation, and remodeling through non-canonical Wnt signaling, while OM stimulated endochondral bone development and mineralization via canonical Wnt3a signaling. These findings highlight the importance of Ti6Al4V surface properties in directing MSC differentiation and indicate that MN-modified surfaces act via signaling pathways that differ from OM culture methods, more accurately mimicking peri-implant osteogenesis in vivo.

## 1. Introduction

Recent studies show that human bone marrow stromal cells (MSCs) exhibit an osteoblast phenotype when they are cultured on titanium and titanium–aluminum–vanadium (Ti6Al4V) substrates that have a surface topography that mimics the macroscale, microscale, and nanoscale features of an osteoclast resorption pit on bone [1,2]. The conditioned media from MSC cultures grown on these Ti6Al4V substrates demonstrate osteoinductive properties, evidenced by their ability to induce bone formation when implanted in muscle as per ASTM F2529-13, the Standard Guide for in vivo Evaluation of Osteoinductive Potential for Materials Containing Demineralized Bone (DBM) [3].

This osteoinductive potential is due, at least in part, to bone morphogenetic protein 2 (BMP2), based on the observation that treatment with anti-BMP2 antibodies abolishes the osteoinductive property of the conditioned medium [4]. However, other factors may also contribute to this osteogenic effect, including semaphorin 3A, which has been shown to stimulate the osteoblast differentiation of MSCs [5] and promote peri-implant osteogenesis in vivo [6].

In addition to producing osteogenic factors, MSCs produce factors that regulate the immune environment [7,8,9]. Earlier studies comparing MSC differentiation on Ti6Al4V substrates that have a surface topography with structural features of an osteoclast resorption pit to machined substrates and polyether–ether–ketone (PEEK) showed that cells on Ti6Al4V generated immune mediators associated with a pro-regenerative response. This effect was greatest on the microstructured Ti6Al4V surface than on the machined surfaces, while cells cultured on PEEK produced immunomodulators indicative of a chronic immune response [10,11].

Osteoblastic differentiation on Ti and Ti6Al4V surfaces with complex microscale topographies has been well characterized [12]. There is a transition from canonical Wnt3a, which promotes proliferation [13], to the production of non-canonical Wnt5a. This shift depends on the production of Wnt11 [14] and a change in integrin expression from alpha5, beta1 (α5β1) to α2β1 [15], which is accompanied by a change in planar cell polarity from a fibroblastic phenotype to a more columnar phenotype [16]. This series of events is very different from what occurs on TCPS, which involves canonical Wnt3a signaling [17].

When MSCs are cultured on biomimetic Ti6Al4V surfaces, osteoblast differentiation occurs within 7 days in standard MSC growth medium (GM) [12]; BMP2 production is increased at this time, reaching peak levels of expression by day 14 [4,18]. In contrast, when MSCs are grown on tissue culture polystyrene (TCPS), osteoblastic differentiation is not observed until day 21 when multi-cellular nodules have formed. Moreover, the expression of an osteoblast phenotype is only consistent when osteogenic media (OM) are used [19].

This difference in MSC behavior under the two culture conditions raises questions concerning the mechanisms involved. Conventionally, laboratories have used OM to ensure the osteoblast differentiation of MSCs. However, the use of OM may result in non-physiological mineral deposition [20,21]. OM contains dexamethasone, which stimulates alkaline phosphatase activity, and as much as 10 mM beta-glycerophosphate (BGP), which is a substrate for alkaline phosphatase [22,23], providing a local source of inorganic phosphate ions needed for calcium phosphate formation. The base medium used to generate OM is also rich in Ca^2+^. Thus, the use of OM and its non-physiological impact arises from the high calcium phosphate ion product generated and not from an organized formation of apatite crystals in the extracellular matrix [20,21]. In contrast, MSCs cultured on microstructured Ti and Ti6Al4V substrates differentiate into osteoblasts without the use of OM [24]. Thus, the use of OM for MSCs grown on biomimetic surfaces can confuse the interpretation of the outcome, as it becomes unclear whether the osteoblast phenotype observed in culture is attributable to surface properties or media composition, and what other differences in cell response might result. This study compares the two culture conditions for the first time, and examines the underlying mechanisms involved by analyzing the signaling pathways involved.

Two Ti6Al4V surfaces, which have been used successfully clinically, were used in the present study. The MN surface, which has microscale and nanoscale surface features, supports the osteoblast differentiation of MSCs and enhances osseointegration in vivo [3,4]. We and others have shown that osteoblast differentiation also occurs on anodized Ti6Al4V [25,26,27]. Similarly, machined Ti6Al4V implants have been used successfully in bone, and MSCs cultured on these substrates produce a pro-healing inflammasome, but to a lesser extent than on microtextured surfaces [11]. A goal of this study was to directly compare MSC responses on Ti6Al4V surfaces that are used clinically: MN vs. anodized vs. smooth machined. A second goal was to directly compare osteoblast differentiation on the MN surface with differentiation on TCPS in OM. We first confirmed that the MN surface would generate a pro-healing inflammasome when compared to an anodized surface we previously demonstrated to have osteogenic effects, and compared to control surfaces with a smooth topography created by machining [26]. We then investigated if the response of MSCs on Ti6Al4V substrates with MN topography is equivalent to osteoblast differentiation by MSCs on TCPS in OM. Finally, we tested whether MSCs on TCPS in OM generate a pro-osteogenic, pro-regenerative immune environment comparable to the microenvironment created by MSCs on Ti6Al4V. The findings generated by these inquiries will provide crucial insights into the role of surface topography and chemistry in modulating MSC behavior, their potential implications for implant design and tissue engineering applications, and whether OM should be used to determine if a material is osteogenic as a function of its surface properties.

## 2. Materials and Methods

### 2.1. Ti6Al4V Disk Fabrication

Grade 23 alloyed Ti6Al4V rods (15 mm diameter) were machined into 1.6 mm thick disks, designed to precisely fit wells in a 24-well plate. Anodized surfaces were prepared using commercial manufacturing methods (Type II anodization, Medtronic Inc., Minneapolis, MN, USA) per ASM 2488, using an alkaline solution with pH 13 or higher. The surface was finished with a glass bead blast. This process resulted in an oxide layer between 1 and 2.5 μm in thickness. Micro/nano (MN)-substrates were grit-blasted and acid-etched using commercial manufacturing methods (nanoLOCK^®^, Medtronic Inc.). All disks were sterilized via gamma irradiation.

### 2.2. Surface Characterization

#### 2.2.1. Scanning Electron Microscopy (SEM)

Surface topography and morphology were qualitatively visualized using scanning electron microscopy (SEM; Hitachi SU-70, Tokyo, Japan). Substrates were mounted on SEM imaging platforms using carbon tape and imaged under the following parameters: a 56 μA ion current, a 5 kV accelerating voltage, and a 5 mm working distance. Six different locations on two separate surfaces were imaged at multiple resolutions.

#### 2.2.2. Contact Angle Analysis

Contact angle measurements were performed using a goniometer (CAM 250, Rame-Hart, Succasunna, NJ, USA) employing the sessile drop method/test with water. Measurements were taken at six distinct locations on two different surfaces (*n* = 12), with the surfaces dried using nitrogen gas between measurements. A 3 μL droplet of distilled water was used for each measurement.

#### 2.2.3. Roughness Analysis

Optical profilometry to measure surface topography was performed using a confocal microscope (Zeiss LSM 710, Carl-Zeiss AG, Oberkochen, Germany). A main beam splitter was set to T80/R20 with reflectance, and Z-stacks were acquired at 1.00 µm intervals using a high pass filter with a cut-off at 20 μm. Measurements were taken at six different locations on two different surfaces (*n* = 12).

#### 2.2.4. Chemical Analysis

##### X-Ray Photoelectron Spectroscopy (XPS)

Elemental composition was analyzed using XPS (PHI VersaProbe III Scanning XPS, Physical Electronics Inc., Chanhassen, MN, USA). Samples were secured to the instrument mount using copper clips, with the mount pre-cleaned via sonication in ethanol solution prior to use. Analysis was performed using a 50-Watt, 15 kV X-ray gun with a 200 µM spot size, 20 ms dwelling time, and 1 eV step size. Two samples per treatment group were analyzed at six different surface positions (*n* = 12).

##### Energy-Dispersive X-Ray (EDX) Analysis

Chemical analyses and elemental mapping of surfaces were conducted using an FE-SEM (Quanta FE-SEM, FEI Co., Hillsboro, OR, USA) equipped with EDX capability. Analyses were conducted using an acceleration voltage of 10 kV and a beam current of 16–48 nA. The data are presented as the mean and standard deviation (SD) of 10 areas measured.

##### X-Ray Diffraction (XRD)

Crystal structure identification was performed using an X’Pert PRO Alpha-1 diffractometer (PANalytical, Almelo, The Netherlands). XRD scans were collected using Cu Kα radiation with a 1° parallel plate collimator, a ½ divergence slit, and a 0.04 rad soller slit for controlled axial divergence. Bragg–Brentano para-focusing at 45 kV and 40 mA was employed. The assignment of detected peaks to crystalline phases was performed using the International Centre for Diffraction Data (ICDD) PDF-4+ reference database.

### 2.3. Response to Surface Topography

#### 2.3.1. Cell Culture

Human bone marrow stromal cells (MSCs) were purchased from Ossium Health (RUO MSC P2). They were isolated from the bone marrow of a 23-year-old white female (batch number 20000127PD). The cells were cultured in MSC growth medium (GM) consisting of αMEM (modified Eagle’s medium) without nucleosides (Gibco Catalog #12561-056, Waltham, MA, USA), supplemented with 2 µM L-glutamine (Life Technologies, Catalog #25030-081, Carlsbad, CA, USA) and 10% fetal bovine serum (Gemini Biproducts, Catalog #900-108, West Sacramento, CA, USA), and 0.5% penicillin/streptomycin (Corning, Catalog #30-002-CI, Corning, NY, USA).

The MSCs were cultured to confluence on TCPS in GM before plating on the test surfaces. Confluent human female MSCs were seeded onto TCPS, smooth machined, anodized, and MN Ti6Al4V surfaces in 24-well plates at a density of 10,000 cells/cm^2^ in 0.5 mL per well (20,000 cells/well). Twelve wells of each type were used per experiment with duplicates, resulting in a sample size of *n* = 6 per group to generate sufficient mRNA for PCR array analysis. The media were changed at 24 h post-seeding and subsequently every 48 h. On day fourteen, fresh media were added to the cultures, and cell layers were lysed and homogenized in TRIzol^®^ for RNA isolation via phenol–chloroform extraction and ethanol precipitation.

#### 2.3.2. RNA Expression Analysis

Homogenized samples in TRIzol^®^ were transferred to pre-chilled, equilibrated phase lock gel (PLG) tubes in 1 mL aliquots. RNA extraction was performed using a modified chloroform-based protocol, followed by isopropanol precipitation and ethanol washing steps. To equilibrate the PLG tubes, they were centrifuged at 12,000× *g* for 30 s at room temperature. 200 μL of chloroform was added to each PLG tube, and the PLG tubes were shaken vigorously by hand for 15 s. The PLG-tubes were then incubated at room temperature for 5 min to allow phase separation. The PLG tubes were centrifuged at 5000× *g* for 5 min at room temperature; the speed was increased to 10,000× *g* and centrifuging continued for an additional 5 min. 500 μL of the aqueous phase of two samples were transferred to the same new 2 mL RNase-free Eppendorf tube. 800 μL of isopropanol was added to the tube, which was shaken by hand for 15 s, and incubated for 10 min at room temperature. RNA was pelleted by centrifugation (16,000× *g*, 10 min, 4 °C). Supernatants were removed, and the RNA pellet was washed with 500 μL ice-cold 70% ethanol and vortexed briefly. RNA samples were pelleted by centrifugation (16,000× *g*, 5 min, 4 °C), and the supernatant was removed and washed with ethanol for a total of two ethanol washes. The pellet was air-dried at room temperature for 5 min by leaving the tube lid open. RNA pellets were dissolved in 100 μL of nuclease-free water and incubated at 37 °C for 5 min, with vortexing performed periodically to solubilize the RNA. The samples were placed immediately on ice.

The extracted RNA was then subjected to DNase treatment to eliminate genomic DNA contamination. Briefly, 100 uL RNA samples were added to 0.3 μL of human RNase inhibitor (40 U/μL) (Sigma Cat. #R2520), 15 μL of 10× DNase I reaction buffer with MgCl_2_, 69.7 μL nuclease-free water, and 15 μL DNase I (1 U/μL). The reaction mixtures were transferred to new, equilibrated PLG tubes, and 400 μL of Ultrapure™ phenol/chloroform/isoamyl alcohol (25:24:1, v/v) (PCI) solution (Invitrogen) was added per sample. The samples were shaken vigorously by hand for 30 s and centrifuged at 14,500× *g* for 5 min at room temperature. The aqueous phase was transferred to a new 1.5 mL tube, and 0.02 volumes of glycogen (5 mg/mL), 0.1 volumes of 3 M NaOAc pH 5.5, and 2.5 volumes of 100% ice-cold ethanol were added to each sample before immediately storing them at −20 °C to precipitate RNA.

RNA was stored at −20 °C for at least 12 h for the robust recovery of total RNA. The RNA was pelleted via centrifugation (14,500× *g*, 30 min, 4 °C). Supernatants were removed, and the RNA pellet washed twice with 500 μL ice-cold 70% ethanol, as before. The pellet was air-dried at room temperature for 5 min by leaving the tube lid open and dissolved in 50 μL of nuclease-free water. The Samples were vortexed for 15 s and placed on ice. RNA was quantified using a Synergy H1 Hybrid Reader Take 3 Spectroscopy instrument with Gen5 v3.08 software (BioTek) and stored at −80 °C. RNA quality was determined by measuring the 260/280 and 260/230 ratios, with values close to 2.0 considered optimal. First-strand cDNA synthesis was performed using a High-Capacity cDNA Reverse Transcription Kit (Applied Biosystems, Waltham, MA, USA) as per the manufacturer’s protocol. Gene-specific forward and reverse DNA oligo qPCR primers were designed and synthesized commercially.

mRNA was measured via qPCR using Power SYBR Green Master Mix (Applied Biosystems) and optimized gene-specific primers. Fold changes to TCPS were normalized to GAPDH in the array using the web-based PCR Array Data Analysis Software (Qiagen, Hilden, Germany). The fold change to TCPS was normalized to housekeeping genes (GAPDH). A no-template control (NTC) and a no-reverse transcriptase control (No-RT control) were included to check for reagent or gDNA contamination.

### 2.4. Growth on Ti6Al4V in GM vs. Growth on TCPS in OM

#### 2.4.1. Cell Culture

Sprague Dawley (SD) rat bone marrow-derived MSCs were purchased from Cyagen (RASMX-01001, OriCell^TM^, Cyagen, Santa Clara, CA, USA) and cultured in GM (RAXMX-90011, Cyagen) on TCPS or MN in 24-well plates at a density of 10,000 cells/cm^2^ for all experiments. The media were changed 24 h after plating and every 48 h thereafter for 7 days. On day 7, cells cultured on Ti6Al4V were incubated with fresh GM, while cells cultured on TCPS were switched from GM to osteogenic media (OM) (RAXMX-90021, Cyagen) for 12 h. A combination of 8 wells from each group constituted *n* = 3 for each experimental condition.

#### 2.4.2. Gene Expression Analysis

To quantify the mRNA levels, cells were plated as described above. At 7 days, the cells were incubated with fresh media for 12 h. mRNA was isolated from the cells using a TRIzol^®^ (Invitrogen, Carlsbad, CA, USA) extraction protocol. RNA quality was assessed by measuring the 260/280 and 260/230 ratios, with values approaching 2.0 considered optimal. RNA quantification was performed using the Synergy H1 Hybrid Reader Take 3 Spectroscopy instrument with Gen5 v3.08 software (BioTek, Shoreline, WA, USA). Subsequently, 2 mg of extracted RNA was submitted to Genewiz (Research Triangle Park, NC, USA) for next-generation RNA sequencing using an Illumina MiSeq platform and approximately 350 M raw paired-end reads per lane.

#### 2.4.3. Bioinformatics Analysis

The read quality of the RNA-seq data was assessed using FASTQC, and reads were aligned to the NCBI Rattus norvegicus annotation release 105 (Rnor_6.0) using STAR. The “Feature Counts” function was employed to determine the number of reads associated with each gene, and differential expression gene analysis was conducted using DESeq2 (1.32.0) in RStudio [28,29,30]. Normalized differentially expressed counts from DESeq2 [31] were used to identify unique and shared genes, represented in Venn diagrams [32], with a threshold of adjusted *p*-value of less than 0.05 and a log fold change of 2. Volcano plots comparing sample groups were generated using the Galaxy web platform, using count data with a significance threshold of 0.05 and a log fold change threshold of 2 [33]. Heat maps were produced using iDEP, an integrated web application, with data samples centered by subtracting the average expression of each gene, and genes centered by subtracting the mean and normalized by dividing by their standard deviation [34].

#### 2.4.4. Functional Enrichment Analysis

Following the calculation of differentially expressed (DE) genes for each pairwise comparison and filtering for significance, pathway enrichment was performed on bone-related-disease-related genes using the MOET database, an online tool for Rat disease enrichment [35]. DE genes for each pairwise comparison were analyzed using the Rat genome assembly GRCr8. The resulting enriched pathways were manually curated to select those relevant to bone disease, and the associated genes of interest were used for downstream functional enrichment analysis. The R package cluster Profiler was used for the functional enrichment analysis, performing a GO Biological Process overrepresentation analysis [36]. Significant pathways were filtered using a Bonferroni-adjusted *p* value < 0.05. Enriched pathways were visualized using barplots for each pairwise comparison of implants. Venn diagrams were created to illustrate overlapping pathways between implants, and were generated using the ggVennDiagram R package [37].

## 3. Results

### 3.1. Surface Characterization and Analysis

**Figure 1:**
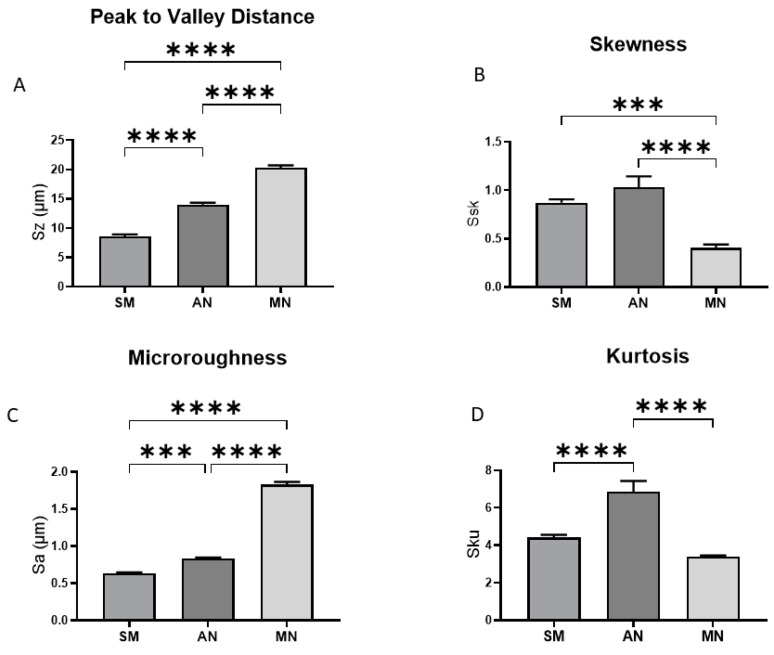
Optical profilometry measurements of surface micro-roughness of smooth machined (SM), anodized (AN), and micro/nano-rough (MN) Ti6Al4V surfaces: average peak-to-valley distance (**A**), skewness (**B**), microroughness (**C**), and kurtosis (**D**). Data are means ± SEM and were evaluated using analysis of variance (ANOVA) with Tukey post hoc test. Statistical significance was established at *p*-values equal to or less than 0.05 (*** *p* < 0.001; **** *p* < 0.0001).

The comparison of the smooth machined Ti6Al4V surface to the anodized and MN modified surfaces demonstrated distinct differences in peak-to-valley distance (Figure 1A) and microroughness (Figure 1B). The smoothed and anodized surfaces exhibited similar skewness, whereas the skewness of the MN surface was lower (Figure 1C). In contrast, the kurtosis of the smooth and MN surfaces was similar, but the kurtosis on the anodized surface was greater than either the smooth or MN surfaces (Figure 1D).

**Figure 2:**
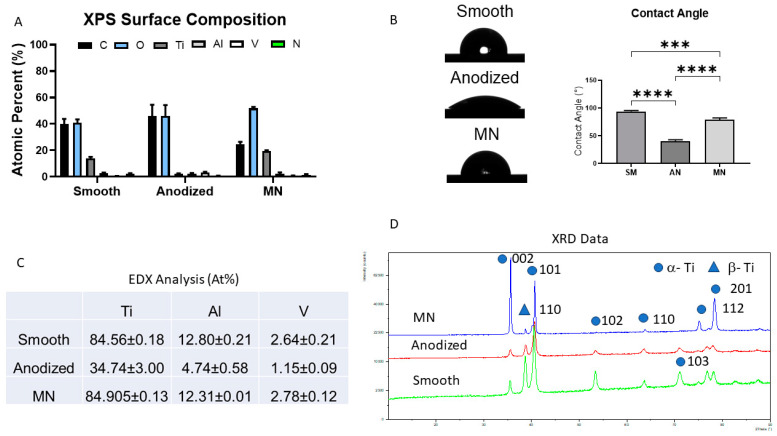
Implant surface characterization of smooth machined (SM), anodized (AN), and micro/nano-rough (MN) Ti6Al4V surfaces: (**A**) X-ray photoelectron spectroscopy (XPS) was used to determine elemental composition, shown as mean atomic percentage (%) of carbon (**C**), oxygen (O), titanium (Ti), aluminum (Al), and vanadium (V). (**B**) Surface wettability was quantified through sessile drop contact angle measurements performed on each surface. (**C**) Semi-quantitative energy-dispersive X-ray spectroscopy (EDX) analysis of surface elemental composition was obtained from ion-milled cross-sections of each surface, shown as mean atomic percentage (%) of titanium (Ti), aluminum (Al), and vanadium (V). (**D**) X-ray diffraction (XRD) profiles were used to determine distribution of α-Ti and β-Ti phases for each surface material, with circles depicting α-Ti, whereas triangles distinguish β-Ti phase based on elemental reference standards for EDX. Data are presented as mean + SEM and were evaluated using analysis of variance (ANOVA) with Tukey post hoc test. Statistical significance was established at *p*-values equal to or less than 0.05 (*** *p* < 0.001; **** *p* < 0.0001).

Surface composition was found to be highly dependent on the fabrication method employed (Figure 2A). Smooth surfaces comprised 40% carbon (C), 40% oxygen (O), and 15% titanium (Ti), while anodized surfaces were predominantly composed of carbon and oxygen. In contrast, the MN surfaces exhibited a composition of 30% C, 50% O, and 20% Ti. Representative XPS Spectra of the three different surfaces are presented in Supplemental Figure S1. These compositional differences were reflected in the contact angle, which followed the order smooth > MN > anodized (Figure 2B). EDX analysis confirmed that both the smooth and MN surfaces maintained a Ti6Al4V composition. The amount of oxygen was below the level of detection (Figure 2C). However, the unique contact angle of the anodized surface suggested the introduction of an additional component during the anodization process. The XRD data revealed peaks for both α-Ti and β-Ti, as would be expected for the Ti6Al4V alloy, demonstrating primarily alpha phase across all surface compositions (Figure 2D). The differences in peak intensity may be attributed to the surface chemistry, oxide thickness, or crystallographic orientation changes that occur in the manufacturing processes to achieve surface texture. Further testing would be required to confirm the contribution of each factor to peak intensity.

**Figure 3:**
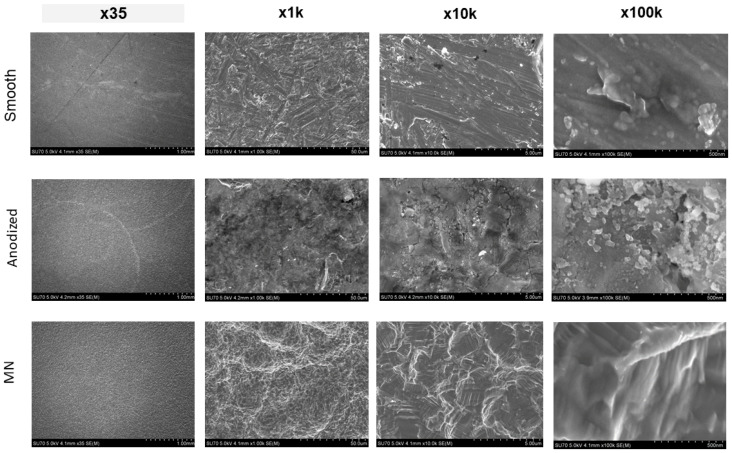
Characterization of surface topography using scanning electron microscopy (SEM). SEM micrographs/images of smooth, anodized, and micro/nano-rough (MN) Ti6Al4V surfaces were captured at macroscale (35×), microscale (1000×), mesoscale (10,000×), and nanoscale (100,000×) resolutions. Macro/micro/meso/nanoscale images have scale bars of 1 mm, 50 μm, 5 μm, and 500 nm, respectively.

SEM imaging of the surfaces showed distinct topographical features specific to each surface treatment (Figure 3).

The smooth surface exhibited randomly oriented machine marks, which were low in height with an area of smooth surface between them. At higher magnification, the surface showed the presence of knob-like projections at the microscale level with no nanoscale structure. The anodized surface had an oxide layer between 1 and 2.5 μm in thickness. It was characterized by a uniform distribution of rounded knobs, approximately 100 nm in diameter. The micro/nano structures were not organized, and the nano structures did not show a defining morphology. In contrast, the MN surface exhibited a complex topography consisting of overlapping microscale pits (approximately 30–50 μm in diameter), microscale scallops (approximately 1–4 μm in diameter), and nanoscale ridges and protrusions superimposed within these microscale features.

### 3.2. MSC Response to Surface Topography

#### 3.2.1. Osteoblast Phenotypic Expression

**Figure 4:**
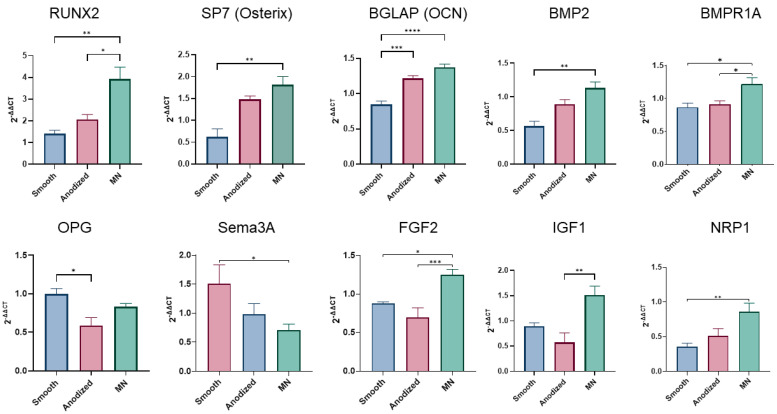
Relative expression of osteogenesis-related genes in human BMSCs cultured for 14 days on TCPS, smooth, anodized, or micro/nano-rough (MN) surfaces via RT-qPCR. Genes were normalized to GAPDH, and their relative expression compared to TCPS, determined using the 2^−ΔΔCT^ method. Values presented are mean ± SE of twelve independent cultures per surface, pooled in duplicate with *n* of 6 per group. Groups were evaluated using analysis of variance (ANOVA) with Tukey post hoc test. Statistical significance was established at *p*-values equal to or less than 0.05 (* *p* < 0.05; ** *p* < 0.01; *** *p* < 0.001; **** *p* < 0.0001).

The mRNA expression of surface-dependent osteoblast differentiation markers in MSCs exhibited a pronounced dependence on surface topography (Figure 4).

The MN surface elicited the highest expression of RUNX2, a key transcription factor in osteogenesis, followed by the anodized and smooth surfaces, respectively. This hierarchical pattern of expression (MN > anodized > smooth) was consistently observed for other important osteogenic markers, including SP7 (Osterix), BGLAP (osteocalcin), BMP2, and BMPR1A. BGLAP expression was elevated on the anodized surface compared to smooth Ti6Al4V, but OPG expression was lowest on the anodized surface, and OPG levels on the MN surface did not surpass those observed on the smooth substrate. The MN surface caused a reduction in semaphorin 3A (sema3A) expression relative to the smooth surface. Conversely, MN surfaces promoted increased expression of the angiogenic factor FGF2, as well as the production of IGF1 and NRP1. Notably, the expression of several other proteins associated with osteogenesis remained unaffected by surface properties (Supplemental Figure S2).

The analysis of oxidative-stress-related genes and transcription factors revealed largely consistent regulation across all substrates, with two exceptions (Supplemental Figure S3). MAPK11 (p38b) was upregulated on MN surfaces, and MAPK9 (JNK2) was downregulated on both anodized and MN substrates (anodized > MN), with a more pronounced effect on the anodized surface. Furthermore, MMP13 expression was significantly upregulated on the MN surface, whereas MMP2, MMP9, MMP14, TIMP1, and TIMP3 remained unaffected by surface modifications (Supplemental Figure S4).

#### 3.2.2. Regulation of Apoptosis

The expression of apoptosis-related genes exhibited significant sensitivity to surface topography (Supplemental Figure S5). The MN surface induced downregulation of several pro-apoptotic genes, including PTGS2 (COX2), RIPK2 (CARD3), TNFRSF1A (p55), AKT1, TNFRSF6 (FAS), and FADD. Conversely, the MN surface promoted the upregulation of HSP90B1 (Hsp90β), CASP1, and THBS1, suggesting complex regulation of apoptotic pathways.

#### 3.2.3. Inflammasome Expression

**Figure 5:**
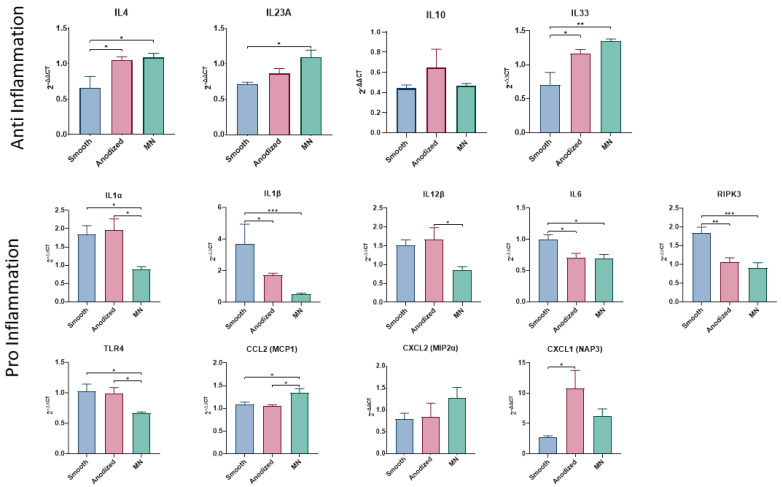
Relative expression of anti-/pro-inflammatory genes in human BMSCs cultured for 14 days on TCPS, smooth, anodized, or micro/nano-rough (MN) surfaces via RT-qPCR. Genes related to innate and adaptive immune mechanisms related to inflammation, including genes that encode for cytokines/chemokines and inflammasome components with anti-inflammatory (top) and pro-inflammatory (bottom) effects, were analyzed. Fold changes to TCPS were normalized to GAPDH and relative expression determined using 2^−ΔΔCT^ method. Values are presented as mean ± SE of twelve independent cultures per surface, pooled in duplicate with *n* of 6 per group. Groups were evaluated using analysis of variance (ANOVA) with Tukey post hoc test. Statistical significance was established at *p*-values equal to or less than 0.05 (* *p* < 0.05; ** *p* < 0.01; *** *p* < 0.001).

Mesenchymal stem cells (MSCs) demonstrated an anti-inflammatory, or pro-healing, inflammasome expression profile when cultured on MN surfaces (Figure 5).

The expression levels of IL4, IL23A, and IL33 were significantly elevated on MN compared to smooth Ti6Al4V, although no difference was observed between MN levels compared to the anodized surface. IL10 expression was unaffected by surface topography. In contrast to their stimulatory effect on anti-inflammatory mediators, MSCs grown on MN surfaces decreased their expression of pro-inflammatory factors, including IL1α, IL1β, IL12β, IL6, RIPK3, and TLR4. The anodized surface similarly reduced expression of IL1β, IL6, and RIPK3 by MSCs. Anodized surfaces similarly reduced the expression of IL1β, IL6, and RIPK3 by MSCs. However, not all pro-inflammatory mediators were downregulated; CCL2 (MCP1) showed increased expression, while CXCL2 remained unaffected. CXCL1 (NAP3) was markedly increased on the anodized surface compared to the smooth surface.

#### 3.2.4. Integrin Expression

**Figure 6:**
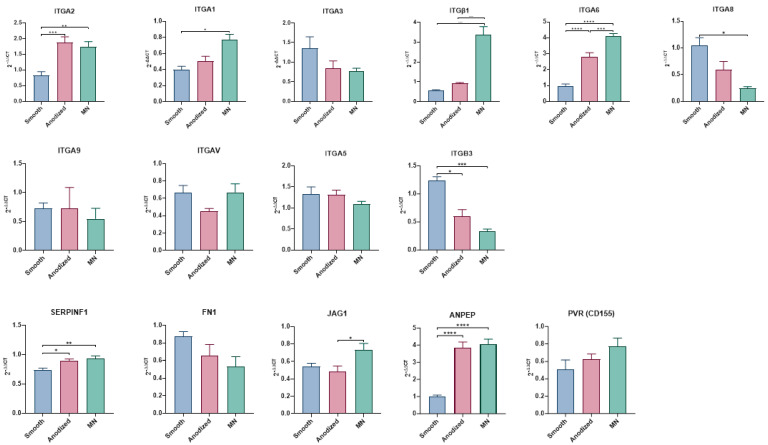
Relative expression of extracellular matrix (ECM)-related genes related to integrin binding and cell adhesion in human BMSCs. Cells were cultured for 14 days on TCPS, smooth, anodized, or micro-/nano-rough (MN) surfaces via RT-qPCR. Representative genes related to integrin binding are on top, and components of ECM are shown in bottom row. Fold changes to TCPS were normalized to GAPDH and relative expression determined using 2^−ΔΔCT^ method. Values are presented as mean ± SE of twelve independent cultures per surface, pooled in duplicate with *n* of 6 per group. Groups were evaluated using analysis of variance (ANOVA) with Tukey post hoc test. Statistical significance was established at *p*-values equal to or less than 0.05 (* *p* < 0.05; ** *p* < 0.01; *** *p* < 0.001; **** *p* < 0.0001).

Integrin expression patterns were sensitive to surface topography (Figure 6). The expression of ITG1, ITGA2, ITG6, and ITGβ1 was upregulated in MSCs grown on MN, with ITGA2 and ITG6 also being increased on anodized surfaces. Conversely, the expression of ITG8 and ITGB3 was reduced on anodized surfaces. The expression of ITGα3, ITG5, ITGA9, and ITGV was unaffected by surface modifications. Other regulatory factors exhibited surface-specific expression patterns. ANPEP and SERPINF1 were comparably elevated on both anodized and MN surfaces, while JAG1 expression increased only on the MN surface. The expression of FN1 and PVR (CD155) remained unaltered across all surface types.

### 3.3. Differential Regulation of Gene Expression and Signaling Pathways in MSCs Cultured on MN-Modified Ti6Al4V Versus TCPS in Osteogenic Media

#### 3.3.1. RNA-seq and Principal Component Analysis

**Figure 7:**
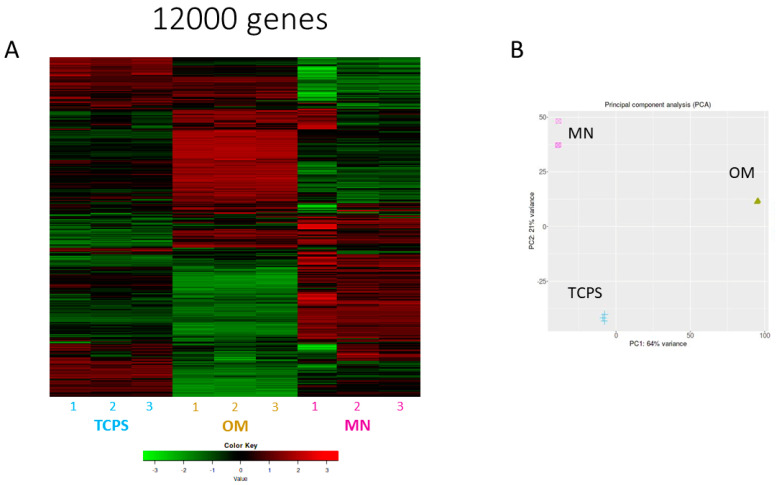
Comparison of 12,000 differentially expressed genes (DEGs) of human BMSCs grown in osteogenic media (OM), compared with BMSCs cultured on micro/nano-rough (MN) surfaces or TCPS in standard growth media: (**A**) Heatmap of sample z-scores clustered using Euclidean distance measurement. Red and green in heat map denote upregulated and downregulated genes, respectively, while gray denotes no significant difference between groups. (**B**) Principal component analysis (PCA) plot generated from 12,000 differentially expressed genes in hBMSCs grown for 14 days on TCPS in OM, TCPS in growth media, or MN surfaces in growth media, as determined by RNA-seq. Results from two independent experiments are shown.

The comprehensive transcriptome analysis revealed distinct gene expression profiles in MSCs cultured on TCPS in OM versus on MN-modified Ti6Al4V in GM (Figure 7 for 12,000 genes and Supplemental Figure S6 for 100 selected genes).

**Figure 8:**
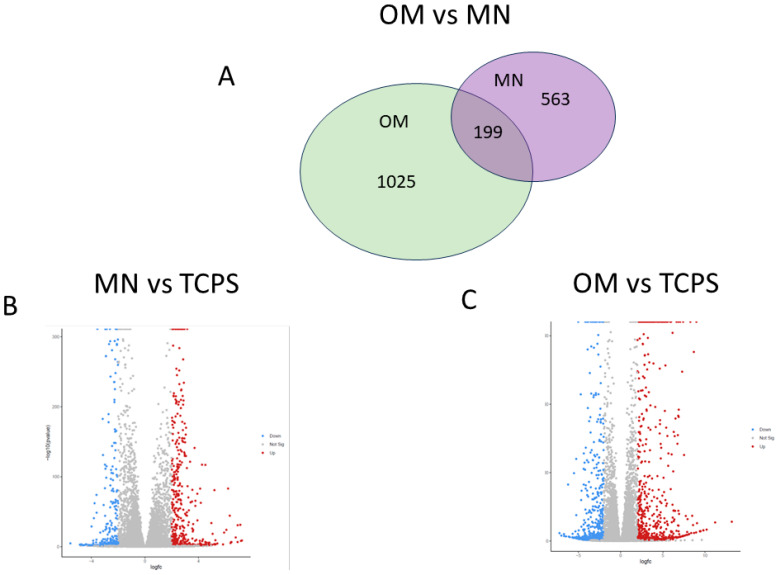
Comparison of 12,000 differentially expressed of genes (DEGs) of human BMSCs grown on TCPS in osteogenic media (OM), or BMSCs cultured on micro/nano-rough (MN) surfaces in growth media, compared to cells on TCPS in growth media. (**A**) Venn diagram displaying overlap of differentially expressed genes, showing overlap between OM and MN cultures. (**B**) Volcano plot of MN vs. TCPS genes with Log2 fold change greater than 2 and adjusted *p*-value less than 0.05. (**C**) Volcano plot of OM vs. TCPS genes with Log2 fold change greater than 2 and adjusted *p*-value less than 0.05. Red and blue denote upregulated and downregulated genes, respectively, while gray denotes no significant difference between groups.

Heat map visualization demonstrated that MSCs seeded on TCPS exhibited a unique gene expression profile when cultured in GM that was distinct from those grown in OM. Notably, MSCs grown on MN surfaces in GM displayed a gene expression profile that diverged from both TCPS culture conditions. The principal component analysis corroborated these findings, elucidating the degree of similarity within each group. Volcano plot comparisons (Figure 8) further emphasized the stark differences in gene expression among the various culture conditions. Quantitatively, MSCs cultured in OM differentially expressed 1025 genes compared to those in GM, while MSCs on MN-modified surfaces showed differential expression of 563 genes relative to TCPS in GM. Remarkably, only 199 genes were commonly regulated between cells cultured in OM and those on MN-modified surfaces.

#### 3.3.2. Pathway Analysis

**Figure 9:**
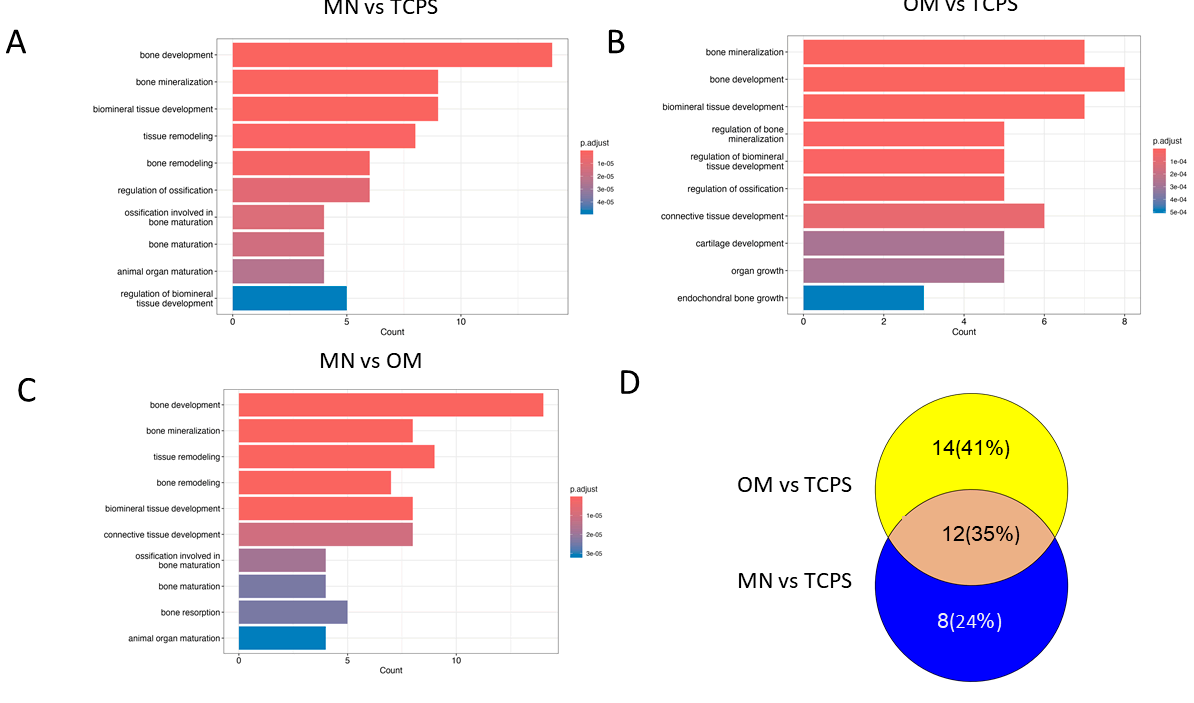
Functional enrichment analysis of genes expressed by BMSCs grown on TCPS in growth media (TCPS), TCPS in osteogenic media (OM), and micro/nano-rough Ti6Al4V in growth media (MN). The enriched pathways were visualized using a barplot for each pairwise comparison of treatments: (**A**) MN vs. TCPS, (**B**) OM vs. TCPS, and (**C**) MN vs. OM. (**D**) The Venn diagrams were created by determining the overlapping pathways between OM vs. TCPS and MN vs. TCPS.

The examination of bone- and cartilage-associated pathways revealed differential emphases in cellular responses across culture conditions (Figure 9).

While bone development emerged as a major outcome in all comparisons, bone mineralization pathways were notably more pronounced in TCPS cultures grown in OM. Additionally, TCPS-OM cultures exhibited enhanced activation of pathways linked to connective tissue and cartilage development, which were not apparent in MSCs cultured on MN-modified surfaces. The comparison of MN vs. TCPS in GM and MSCs on TCPS in OM shows that the canonical Wnt pathway is shared through the expression of Frizzled, LRP 5/6, and REPO, but then diverges with cells on MN expressing ICAT, P53, and sphingosine-1-phosphate (SIP), a bioactive lipid mediator, and cells on TCPS in OM favoring the expression of c-JUN, an integral component of the AP-1 transcription factor complex (Table 1).

Wnt11-mediated planar cell polarity was evident in both OM and MN conditions. However, alternate middle steps in MN cultures enhanced the expression of Prickle, a core component of the planar cell polarity pathway, and Rock2, a key regulator of cytoskeletal reorganization. Conversely, OM conditions resulted in the suppression of their expression. Non-canonical signaling at the receptor level was comparable across culture conditions, although notable distinctions emerged in the downstream pathway mediated through phospholipase C (PLC) and protein kinase C (PKC). PLC and PKC were stimulated in MN cultures but were inhibited under OM conditions.

## 4. Discussion

This study underscores the sensitivity of MSCs to the topographical and chemical attributes of the Ti6Al4V substrates commonly used for implants in vivo. This sensitivity is reflected in a multifaceted cellular response, encompassing alterations in osteoblastic differentiation, the modulation of inflammasome activation, and the reconfiguration of integrin-mediated surface interactions. The surface properties also impact downstream cellular responses, including autocrine and paracrine signaling, extracellular matrix modification, and apoptotic machinery. The observed cellular plasticity in response to these varied substrate characteristics highlights the critical role of the extracellular microenvironment in dictating stem cell fate and function and, ultimately, clinical outcome.

We have previously shown the importance of surface topography in determining osteoblast differentiation on Ti and Ti6Al4V substrates. In general, MSCs exhibit a more osteoblastic phenotype when grown on macro/micro/nanoscale surfaces, with features including 1–3 mm levels of roughness, the morphology of an osteoclast-resorption pit (30 to 100 µm in diameter), and nanoscale to mesoscale roughness that has low skewness and kurtosis, akin to an isosceles triangle shape with a sharp edge [4]. All three Ti6Al4V substrates used in this study have microscale surface features, but only the MN modification has a specifically engineered texture that includes the preferred topography. Notably, anodized substrates induce a more pronounced pro-osteogenic phenotype compared to smooth machined Ti6Al4V, but to a lesser extent than the micro/nano-textured MN surface.

Surface composition analysis via XPS and EDX reveals variations that may influence cellular responses. Differences in surface hydrophobicity likely affect protein and lipid adsorption from the media, subsequently impacting downstream cell attachment and adhesion [38,39,40,41]. Anodized Ti6Al4V surfaces had the highest carbon content and lowest contact angle among the surfaces tested, suggesting that the involvement of factors beyond carbon content may account for differences in surface properties and cellular response. The X-ray diffraction results of the three surfaces were similar, but not identical, further supporting the conclusion that the adsorption of media components differs between surfaces, consequently impacting cell attachment, adhesion, and differentiation.

This investigation corroborates and extends upon our previous findings regarding the modulation of integrin expression during the osteoblastic differentiation of MSCs. As noted in our previous studies, the osteoblast differentiation of MSCs is accompanied by a shift in integrin expression from α5β1 to α2β1 and α1β1 [42,43,44]. Consistent with these observations, this study demonstrates a characteristic shift in the integrin expression profile of MSCs. Specifically, we observed significant upregulation of the integrin subunits ITGα1, ITGα2, and ITGβ1, aligning with the downregulation of ITGβ3. This alteration in integrin expression suggests reconfiguration of the mechanisms involved in cell adhesion, including cell–extracellular matrix interactions.

The expression levels of ITGα5 and ITGαV remained largely unaffected, indicating the selective modulation of specific integrin subunits rather than global alteration of the entire repertoire of integrin subunits. The observed downregulation of ITGβ3, in conjunction with the stable expression of ITGα5 and ITGαV, implies a potential shift in the affinity of cellular adhesion molecules towards collagen type I, a primary constituent of the bone extracellular matrix. Conversely, this integrin expression profile suggests a potential reduction in or the maintenance of cellular interactions with fibronectin and vimentin, two extracellular matrix proteins associated with less differentiated mesenchymal phenotypes. This selective modulation of integrin expression illustrates the complex and precisely regulated nature of cell–matrix interactions during osteoblastic differentiation, providing insights into the molecular mechanisms governing MSCs’ commitment to the osteoblastic lineage and role during osseointegration.

We demonstrate for the first time that the growth of MSCs on MN surfaces is associated with the upregulation of integrin α6 (ITGA6), a receptor known to interact with extracellular matrix proteins such as fibronectin and laminin family members [45]. ITGα6 has been implicated in cellular adhesion, migration, and epithelial-to-mesenchymal transition processes [46], although its precise role in mediating MSCs’ responses to MN surface textures remains to be elucidated.

We also observed downregulation of mRNA for integrin α8 (ITGA8). ITGα8 functions as a receptor for tenascin, fibronectin, and vitronectin [47], which is important for MSC attachment and adhesion to the implant surface. The α8β1 integrin pair also binds nephronectin, an extracellular matrix protein, which is involved in the osteoblast differentiation of osteoprogenitor cells [48]. The concomitant increase in ITGα6 and decrease in ITGα8 expression as the cells shift from migration and proliferation to extracellular matrix synthesis and attachment to type 1 collagen supports the hypothesis that MN surfaces promote osteoblastic differentiation rather than a proliferative fibroblastic phenotype.

The observed shift in integrin expression is notably accompanied by the concomitant upregulation of BMP2 and its cognate receptor, BMPR1A. This supports our previous data, reinforcing the notion that cells not only produce this crucial autocrine regulator of osteoblast differentiation but are also receptive to it [4]. The functional significance of this BMP2-BMPR1A axis is highlighted by inhibition studies, wherein the blockade of either BMP2 or its receptor abrogates the surface topography-induced osteoblastic differentiation and attenuates the osteoinductive capacity of conditioned media derived from these cultures.

Further substantiating the osteogenic differentiation process on MN surfaces and, to a lesser extent, on anodized surfaces is the observed upregulation of RUNX2 and SP7 (osterix), which are transcription factors that serve as master regulators orchestrating the cellular differentiation program during osteogenesis. RUNX2 functions as an early-stage transcriptional activator, while osterix acts as a late-stage transcription factor, collectively governing the sequential progression of osteoblastic differentiation [49,50]. The functional consequence of enhanced RUNX2 and SP7 expression is manifested in the increased transcription of BGLAP, which encodes osteocalcin, a non-collagenous protein abundant in the bone matrix, which serves as a definitive marker of mature osteoblasts and plays a crucial role in calcium homeostasis and bone mineralization [51].

The cascade of molecular events involved in integrin modulation, the enhancement of BMP2 signaling, and the activation of osteogenic transcription factors collectively delineate a comprehensive mechanism by which surface topography influences mesenchymal stem cell fate decisions, directing them towards the osteoblastic lineage. These findings not only enhance our understanding of the molecular mechanisms of surface-induced osteogenesis, but also provide valuable insights for the rational design of biomaterials aimed at enhancing osseointegration and bone regeneration in clinical applications.

Elevated levels of pro-angiogenic factors like FGF2, NRP1, and IGF1 in MSCs grown on MN surfaces indicate enhanced potential for bone regeneration and osteointegration. Specifically, we observed an increase in FGF2, a potent angiogenic factor that promotes blood vessel formation. Concurrently, there was an elevation in neuropilin-1 (NRP1), a co-receptor for VEGF, which plays an important role in regulating vascular development. Additionally, IGF1, which facilitates normal bone and tissue growth and development, was also found to be upregulated. Our observations suggest that these factors are regulated in a reciprocal manner. The collective action of these factors creates a microenvironment conducive to neovascularization and tissue growth, both of which are critical for successful implant integration and long-term stability.

Our study revealed a nuanced interplay between various osteogenic factors. While BMP2 and its receptor exhibited increased expression on MN surfaces, sema3A levels were concomitantly reduced. This observation is particularly intriguing given that sema3A, like BMP2, has been demonstrated to stimulate osteoblast differentiation in vitro [6] and promote peri-implant bone formation in vivo [52].

The reciprocal regulation of BMP2 and sema3A suggests a complex, finely tuned mechanism governing osteoblast differentiation and bone formation in response to MN surface topography. This inverse relationship may represent a homeostatic mechanism that optimizes the osteogenic response while preventing excessive bone formation. Alternatively, it could indicate a temporal sequence in the activation of these factors during the process of osteogenesis and implant integration. Such insights have profound implications for the design and optimization of implant surfaces to direct angiogenesis to maximize osseointegration and long-term implant success in clinical applications.

Our investigation reveals that certain molecular factors are consistently expressed under different culture conditions. These factors include BMP4, BMPR2, TGFβ1, TGFβ2, TGFβR1, sema3C, VEGFA, ANG1, and NRP2, among others. It is important to note that this analysis represents a snapshot in time, making it difficult to interpret these findings in the overall context of the cell response to the surface. Our results demonstrate, however, that cellular responses are highly sensitive to the micro/nano surface topography and chemistry. Moreover, we focused our analysis on genes encoding proteins integral to bone tissue regeneration, including cell recruitment, attachment, and proliferation. Specifically, we observed the modulation of several key genes, including ANPEP, encoding alanyl aminopeptidase, a membrane-bound enzyme involved in peptide catabolism; FN1, encoding fibronectin, an extracellular matrix glycoprotein crucial for cell adhesion; JAG1, encoding a protein pivotal for new blood vessel development; SERPINF1, known for its dual role as an anti-angiogenic factor and neurotrophic agent; and PVR, a protein implicated in cell proliferation and migration. Of particular interest is the coordinated upregulation of ANPEP, JAG1, and SERPINF1 observed on the MN surface. This specific gene expression profile suggests that cells on the MN surface are actively modifying their extracellular milieu to promote vascularization and neurogenesis; such processes are intrinsically linked to successful bone regeneration. These findings collectively indicate that MN surface modifications elicit a sophisticated cellular response, characterized by the orchestrated regulation of genes involved in tissue remodeling, angiogenesis, and neurogenesis.

The observed cellular responses to micro/nano surface modifications are further corroborated by alterations in the inflammasome profile generated by MSCs cultured on these surfaces. Notably, there is marked upregulation of anti-inflammatory mediators, including IL4, IL23A, and IL33, concomitant with the downregulation of pro-inflammatory factors. While similar changes are observed on anodized surfaces, they occur to a much lesser extent.

Interestingly, the expression of IL10 remained unaffected by surface topography and chemistry in the present study. This contrasts with earlier investigations comparing MSC responses to smooth Ti6Al4V and grit-blasted/acid-etched surfaces with macro/micro/nanoscale (MMN) features, which demonstrated IL10 upregulation on MMN surfaces relative to PEEK [11]. This discrepancy suggests that IL10 production may be mediated by larger-scale topographical features or represents an early-stage event in the cellular response.

This study also revealed upregulation of chemokine (C-C motif) ligand 2 (CCL2) on MN surfaces, a factor known to recruit monocytes, memory T cells, and dendritic cells to sites of inflammation following tissue injury. Concurrently, chemokine (C-X-C motif) ligand 1 (CXCL1), which facilitates neutrophil recruitment and activation for microbial elimination, was upregulated on anodized surfaces. Additionally, Toll-like receptor 4 (TLR4), a key sensor for Gram-negative lipopolysaccharides (LPS) and other infectious stimuli, showed increased expression. These findings collectively suggest that MSCs cultured on MN surfaces promote the recruitment of immune cells that enhance regeneration while modulating factors and receptors involved in infection response.

Importantly, this study shows for the first time that the differentiation process on MN occurs through mechanisms distinct from those observed in MSCs cultured in osteogenic media. This disparity raises questions regarding the applicability of defining nanoscale features as osteogenic based solely on OM culture conditions. RNA-sequencing analysis revealed marked differences between MN and OM culture conditions, with only 199 genes shared between the two and only a 35% overlap in activated pathways. Notably, OM stimulates pathways involved in endochondral bone development, a process that was not observed in MN cultures. Conversely, MN surfaces activate pathways associated with bone formation, maturation, and remodeling, while OM predominantly stimulates mineralization-related pathways. The validation of the results of the RNA-seq was found in the gene expressed in MSCs grown on the MN surface compared to the smooth Ti6Al4V surface presented in Figure 4 (genes involved in osteogenic differentiation) and Figure 6 (integrin genes).

The divergent mechanisms of osteogenic induction are further exemplified by the activation of canonical Wnt3a signaling in OM cultures, contrasting with the non-canonical Wnt signaling pathways observed in MN cultures. Both conditions result in alterations in planar cell polarity but through distinct molecular routes. This observation aligns with our previous findings, demonstrating that MMN and MN surfaces promote a shift from canonical Wnt3A signaling to non-canonical Wnt5A signaling, whereas cells cultured on TCPS in OM differentiate via Wnt3A.

Changes in the inflammasome generated by cells on MN surfaces, characterized by the upregulation of anti-inflammatory mediators and downregulation of pro-inflammatory factors, further support the pro-regenerative potential of these surfaces. The differential regulation of chemokines and immune receptors suggests that MN surfaces have the capacity to modulate the inflammatory response to potentially promote enhanced bone regeneration.

## 5. Conclusions

Collectively, these findings emphasize the complex and multifaceted nature of surface-induced osteogenesis, highlighting the importance of considering both surface properties and culture conditions when evaluating the osteogenic potential of biomaterials. The distinct molecular mechanisms underlying MN-induced osteogenesis suggest that these surfaces may better mimic the in vivo osteogenic microenvironment compared to traditional culture systems that use osteogenic media. This paper reports for the first time a direct comparison of MSC osteoblastic differentiation on microscale/nanoscale rough TiAl6V4 surfaces with MSCs that are cultured in “osteogenic media”. The data demonstrate clearly that the two processes are very different. Given that OM is used in the bone field to induce “osteogenesis”, these data are critically important to the understanding of what they are actually measuring and what relationship it has, if any, with the actual peri-implant events that occur in vivo. This finding carries significant implications for the field of regenerative medicine and the rational design of biomaterials, as it suggests that MN-modified surfaces possess the potential to confer substantial advantages in clinical applications aimed at enhancing bone regeneration and promoting robust implant integration.

## Figures and Tables

**Table 1:** Comparison of differentially expressed genes (DEGs) in the canonical Wnt signaling pathway, the noncanonical Wnt pathway (Wnt/Ca), and the planar cell polarity pathway. Human BMSCs were grown on TCPS in osteogenic media (OM) or on micro/nano-rough (MN) surfaces in growth media. Their expression was compared to BMSCs cultured on TCPS in growth media. Red text shows increased expression; green text shows inhibition of expression; and yellow highlighting indicates changes in expression shared by both culture conditions.

**Canonical Pathway**															
MN vs. TCPS	RSPO	FRP	CycD	Frizzled	LRP5/6	Notum	AXIM	BAMBI	P53	ICAT	GBP	APC	PRARD	CK2	SIP
OM vs. TCPS	RSPO	FRP	CycD	Frizzled	LRP5/6	Notum	AXIM	BAMBI	P53	ICAT	APC	PKA	NKD	Catenin	C-JUN
**Planar Cell Polarity**															
MN vs. TCPS	WNT11	Frizzled	Daam1	ROR1/2	Knypek	Prickle	ROCK2								
OM vs. TCPS	WNT11	Frizzled	Daam1	DVL	JNK	Prickle	ROCK2								
**Wnt/Ca (Non-Canonical Pathway)**															
MN vs. TCPS	Frizzled	CaMKII	NFAT	PLC	PKC										
OM vs. TCPS	Frizzled	CaMKII	NFAT	PLC	PKC										

## Data Availability

The raw data required to reproduce these findings are available upon reasonable request from the corresponding author. The processed data required to reproduce these findings are available upon reasonable request from the corresponding author.

## Supplementary Materials

The following supporting information can be downloaded at: https://www.mdpi.com/article/10.3390/biomimetics10010066/s1, Supplementary Figure S1: Representative XPS Spectra of the three different surfaces used throughout the studies; Supplementary Figure S2: Relative expression of osteogenesis- and angiogenesis-related genes in human BMSCs cultured for 14 days on TCPS, smooth, anodized, or micro/nano-rough (MN) surfaces via RT-qPCR; Supplementary Figure S3: Relative expression of oxidative-stress-related and transcription factors and related genes in human BMSCs cultured for 14 days on TCPS, smooth, anodized, or micro/nano-rough (MN) surfaces via RT-qPCR; Supplementary Figure S4: Relative expression of extracellular matrix processing genes in human BMSCs cultured for 14 days on TCPS, smooth, anodized, or micro/nano-rough (MN) surfaces via RT-qPCR; Supplementary Figure S5: Relative expression of pro- and anti-apoptosis-related genes in human BMSCs cultured for 14 days on TCPS, smooth, anodized, or micro/nano-rough (MN) surfaces via RT-qPCR; Supplementary Figure S6: Comparison of differentially expressed genes (DEGs) from a panel of 100 genes associated with osteogenesis. Human BMSCs were grown for 14 days on TCPS in osteogenic media (OM), on TCPS in growth media (TCPS), or on micro/nano-rough Ti6Al4V surfaces (MN) in growth media.

## References

[1] Berger M.B., Slosar P., Schwartz Z., Cohen D.J., Goodman S.B., Anderson P.A., Boyan B.D. (2022). A Review of Biomimetic Topographies and Their Role in Promoting Bone Formation and Osseointegration: Implications for Clinical Use. Biomimetics.

[2] Liu T., Pang Y., Zhou Z., Yao R., Sun W. (2019). An Integrated Cell Printing System for the Construction of Heterogeneous Tissue Models. Acta Biomater..

[3] Berger M.B., Cohen D.J., Bosh K.B., Kapitanov M., Slosar P.J., Levit M.M., Gallagher M., Rawlinson J.J., Schwartz Z., Boyan B.D. (2023). Bone Marrow Stromal Cells Generate an Osteoinductive Microenvironment When Cultured on Titanium–Aluminum–Vanadium Substrates with Biomimetic Multiscale Surface Roughness. Biomed. Mater..

[4] Berger M.B., Bosh K.B., Jacobs T.W., Joshua Cohen D., Schwartz Z., Boyan B.D. (2021). Growth Factors Produced by Bone Marrow Stromal Cells on Nanoroughened Titanium–Aluminum–Vanadium Surfaces Program Distal MSCs into Osteoblasts via BMP2 Signaling. J. Orthop. Res..

[5] Lotz E.M., Berger M.B., Boyan B.D., Schwartz Z. (2020). Regulation of Mesenchymal Stem Cell Differentiation on Microstructured Titanium Surfaces by Semaphorin 3A. Bone.

[6] Deng J., Cohen D.J., Berger M.B., Sabalewski E.L., McClure M.J., Boyan B.D., Schwartz Z. (2023). Osseointegration of Titanium Implants in a Botox-Induced Muscle Paralysis Rat Model Is Sensitive to Surface Topography and Semaphorin 3A Treatment. Biomimetics.

[7] Pittenger M.F., Discher D.E., Péault B.M., Phinney D.G., Hare J.M., Caplan A.I. (2019). Mesenchymal Stem Cell Perspective: Cell Biology to Clinical Progress. NPJ Regen. Med..

[8] Müller L., Tunger A., Wobus M., von Bonin M., Towers R., Bornhäuser M., Dazzi F., Wehner R., Schmitz M. (2021). Immunomodulatory Properties of Mesenchymal Stromal Cells: An Update. Front. Cell Dev. Biol..

[9] Smolinská V., Boháč M., Danišovič Ľ. (2023). Current Status of the Applications of Conditioned Media Derived from Mesenchymal Stem Cells for Regenerative Medicine. Physiol. Res..

[10] Olivares-Navarrete R., Hyzy S.L., Berg M.E., Schneider J.M., Hotchkiss K., Schwartz Z., Boyan B.D. (2014). Osteoblast Lineage Cells Can Discriminate Microscale Topographic Features on Titanium–Aluminum–Vanadium Surfaces. Ann. Biomed. Eng..

[11] Olivares-Navarrete R., Hyzy S.L., Slosar P.J., Schneider J.M., Schwartz Z., Boyan B.D. (2015). Implant Materials Generate Different Peri-Implant Inflammatory Factors. Spine.

[12] Boyan B.D., Berger M.B., Nelson F.R., Donahue H.J., Schwartz Z. (2022). The Biological Basis for Surface-Dependent Regulation of Osteogenesis and Implant Osseointegration. J. Am. Acad. Orthop. Surg..

[13] Boland G.M., Perkins G., Hall D.J., Tuan R.S. (2004). Wnt 3a Promotes Proliferation and Suppresses Osteogenic Differentiation of Adult Human Mesenchymal Stem Cells. J. Cell Biochem..

[14] Boyan B.D., Olivares-Navarrete R., Berger M.B., Hyzy S.L., Schwartz Z. (2018). Role of Wnt11 during Osteogenic Differentiation of Human Mesenchymal Stem Cells on Microstructured Titanium Surfaces. Sci. Rep..

[15] Olivares-Navarrete R., Hyzy S.L., Park J.H., Dunn G.R., Haithcock D.A., Wasilewski C.E., Boyan B.D., Schwartz Z. (2011). Mediation of Osteogenic Differentiation of Human Mesenchymal Stem Cells on Titanium Surfaces by a Wnt-Integrin Feedback Loop. Biomaterials.

[16] Lai M., Hermann C.D., Cheng A., Olivares-Navarrete R., Gittens R.A., Bird M.M., Walker M., Cai Y., Cai K., Sandhage K.H. (2015). Role of A2β1 Integrins in Mediating Cell Shape on Microtextured Titanium Surfaces. J. Biomed. Mater. Res. A.

[17] Jullien N., Maudinet A., Leloutre B., Ringe J., Häupl T., Marie P.J. (2012). Downregulation of ErbB3 by Wnt3a Contributes to Wnt-induced Osteoblast Differentiation in Mesenchymal Cells. J. Cell Biochem..

[18] Velazquez-Cayon R., Castillo-Dali G., Corcuera-Flores J., Serrera-Figallo M., Castillo-Oyague R., Gonzalez-Martin M., Gutierrez-Perez J., Torres-Lagares D. (2017). Production of Bone Mineral Material and BMP-2 in Osteoblasts Cultured on Double Acid-Etched Titanium. Med. Oral. Patol. Oral. Cir. Bucal.

[19] Vater C., Kasten P., Stiehler M. (2011). Culture Media for the Differentiation of Mesenchymal Stromal Cells. Acta Biomater..

[20] Boyan B.D., Bonewald L.F., Paschalis E.P., Lohmann C.H., Rosser J., Cochran D.L., Dean D.D., Schwartz Z., Boskey A.L. (2002). Osteoblast-Mediated Mineral Deposition in Culture Is Dependent on Surface Microtopography. Calcif. Tissue Int..

[21] Bonewald L.F., Harris S.E., Rosser J., Dallas M.R., Dallas S.L., Camacho N.P., Boyan B., Boskey A. (2003). Von Kossa Staining Alone Is Not Sufficient to Confirm That Mineralization In Vitro Represents Bone Formation. Calcif. Tissue Int..

[22] Leboy P.S., Beresford J.N., Devlin C., Owen M.E. (1991). Dexamethasone Induction of Osteoblast MRNAs in Rat Marrow Stromal Cell Cultures. J. Cell. Physiol..

[23] Volk S.W., Diefenderfer D.L., Christopher S.A., Haskins M.E., Leboy P.S. (2005). Effects of Osteogenic Inducers on Cultures of Canine Mesenchymal Stem Cells. Am. J. Vet. Res..

[24] Wang Q., Wang X., Valverde P., Murray D., Dard M.M., Van Dyke T., Xu Q., Xu X., Karimbux N., Tu Q. (2021). Osteogenic Effects of MicroRNA-335-5p/Lipidoid Nanoparticles Coated on Titanium Surface. Arch. Oral. Biol..

[25] Nahum E.Z., Lugovskoy A., Lugovskoy S., Sobolev A. (2023). Synthesis of Titanium Oxide Nanotubes Loaded with Hydroxyapatite. Nanomaterials.

[26] Zhao G., Zinger O., Schwartz Z., Wieland M., Landolt D., Boyan B.D. (2006). Osteoblast-like Cells Are Sensitive to Submicron-scale Surface Structure. Clin. Oral. Implants Res..

[27] Berthelot R., Variola F. (2025). Investigating the Interplay between Environmental Conditioning and Nanotopographical Cueing on the Response of Human MG63 Osteoblastic Cells to Titanium Nanotubes. Biomater. Sci..

[28] Andrés-León E., Núñez-Torres R., Rojas A.M. (2016). MiARma-Seq: A Comprehensive Tool for MiRNA, MRNA and CircRNA Analysis. Sci. Rep..

[29] Robinson M.D., McCarthy D.J., Smyth G.K. (2010). EdgeR: A Bioconductor Package for Differential Expression Analysis of Digital Gene Expression Data. Bioinformatics.

[30] R Core Team (2019). R: A Language and Environment for Statistical Computing.

[31] Love M.I., Huber W., Anders S. (2014). Moderated Estimation of Fold Change and Dispersion for RNA-Seq Data with DESeq2. Genome Biol..

[32] Chen H., Boutros P.C. (2011). VennDiagram: A Package for the Generation of Highly-Customizable Venn and Euler Diagrams in R. BMC Bioinform..

[33] Abueg L.A.L., Afgan E., Allart O., Awan A.H., Bacon W.A., Baker D., Bassetti M., Batut B., Bernt M., Blankenberg D. (2024). The Galaxy Platform for Accessible, Reproducible, and Collaborative Data Analyses: 2024 Update. Nucleic Acids Res..

[34] Ge S.X., Son E.W., Yao R. (2018). IDEP: An Integrated Web Application for Differential Expression and Pathway Analysis of RNA-Seq Data. BMC Bioinform..

[35] Vedi M., Nalabolu H.S., Lin C.-W., Hoffman M.J., Smith J.R., Brodie K., De Pons J.L., Demos W.M., Gibson A.C., Hayman G.T. (2022). MOET: A Web-Based Gene Set Enrichment Tool at the Rat Genome Database for Multiontology and Multispecies Analyses. Genetics.

[36] Yu G., Wang L.-G., Han Y., He Q.-Y. (2012). ClusterProfiler: An R Package for Comparing Biological Themes Among Gene Clusters. OMICS.

[37] Gao C.-H., Yu G., Cai P. (2021). GgVennDiagram: An Intuitive, Easy-to-Use, and Highly Customizable R Package to Generate Venn Diagram. Front. Genet..

[38] Barberi J., Spriano S. (2021). Titanium and Protein Adsorption: An Overview of Mechanisms and Effects of Surface Features. Materials.

[39] Park J.H., Schwartz Z., Olivares-Navarrete R., Boyan B.D., Tannenbaum R. (2011). Enhancement of Surface Wettability via the Modification of Microtextured Titanium Implant Surfaces with Polyelectrolytes. Langmuir.

[40] Gittens R.A., Olivares-Navarrete R., Cheng A., Anderson D.M., McLachlan T., Stephan I., Geis-Gerstorfer J., Sandhage K.H., Fedorov A.G., Rupp F. (2013). The Roles of Titanium Surface Micro/Nanotopography and Wettability on the Differential Response of Human Osteoblast Lineage Cells. Acta Biomater..

[41] Gonzalez Solveyra E., Thompson D.H., Szleifer I. (2022). Proteins Adsorbing onto Surface-Modified Nanoparticles: Effect of Surface Curvature, PH, and the Interplay of Polymers and Proteins Acid–Base Equilibrium. Polymers.

[42] Olivares-Navarrete R., Raz P., Zhao G., Chen J., Wieland M., Cochran D.L., Chaudhri R.A., Ornoy A., Boyan B.D., Schwartz Z. (2008). Integrin A2β1 Plays a Critical Role in Osteoblast Response to Micron-Scale Surface Structure and Surface Energy of Titanium Substrates. Proc. Nat. Acad. Sci. USA.

[43] Olivares-Navarrete R., Rodil S.E., Hyzy S.L., Dunn G.R., Almaguer-Flores A., Schwartz Z., Boyan B.D. (2015). Role of Integrin Subunits in Mesenchymal Stem Cell Differentiation and Osteoblast Maturation on Graphitic Carbon-Coated Microstructured Surfaces. Biomaterials.

[44] Raines A.L., Berger M.B., Schwartz Z., Boyan B.D. (2019). Osteoblasts Grown on Microroughened Titanium Surfaces Regulate Angiogenic Growth Factor Production through Specific Integrin Receptors. Acta Biomater..

[45] Wang T., Zhang W., Fang C., Wang N., Zhuang Y., Gao S. (2024). Research on the Regulatory Mechanism of Ginseng on the Tumor Microenvironment of Colorectal Cancer Based on Network Pharmacology and Bioinformatics Validation. Curr. Comput. Aided Drug Des..

[46] Lee C., Lee C., Lee S., Siu A., Ramos D.M. (2014). The Cytoplasmic Extension of the Integrin Β6 Subunit Regulates Epithelial-to-Mesenchymal Transition. Anticancer Res..

[47] Schnapp L.M., Hatch N., Ramos D.M., Klimanskaya I.V., Sheppard D., Pytela R. (1995). The Human Integrin A8β1 Functions as a Receptor for Tenascin, Fibronectin, and Vitronectin. J. Biol. Chem..

[48] Kinoshita M., Yamada A., Sasa K., Ikezaki K., Shirota T., Kamijo R. (2021). Phorbol-12-Myristate 13-Acetate Inhibits Nephronectin Gene Expression via Protein Kinase C Alpha and c-Jun/c-Fos Transcription Factors. Sci. Rep..

[49] Komori T. (2024). Regulation of Skeletal Development and Maintenance by Runx2 and Sp7. Int. J. Mol. Sci..

[50] Komori T. (2020). Molecular Mechanism of Runx2-Dependent Bone Development. Mol. Cells.

[51] Kim Y.-J., Kim H.-N., Park E.-K., Lee B.-H., Ryoo H.-M., Kim S.-Y., Kim I.-S., Stein J.L., Lian J.B., Stein G.S. (2006). The Bone-Related Zn Finger Transcription Factor Osterix Promotes Proliferation of Mesenchymal Cells. Gene.

[52] Deng J., Cohen D.J., Sabalewski E.L., Van Duyn C., Wilson D.S., Schwartz Z., Boyan B.D. (2023). Semaphorin 3A Delivered by a Rapidly Polymerizing Click Hydrogel Overcomes Impaired Implant Osseointegration in a Rat Type 2 Diabetes Model. Acta Biomater..

