# Supplementary material for: Bone Marrow Stromal Cells Generate a Pro-Healing Inflammasome When Cultured on Titanium–Aluminum–Vanadium Surfaces with Microscale/Nanoscale Structural Features
Source: Biomimetics (Basel). 2025 Jan 19;10(1):66. doi: 10.3390/biomimetics10010066 (PMC11759188; doi:10.3390/biomimetics10010066)
Supplement: Supplementary file 1 [file biomimetics-10-00066-s001.zip › biomimetics-3398784-supplementary.pdf]

Supplemental Data

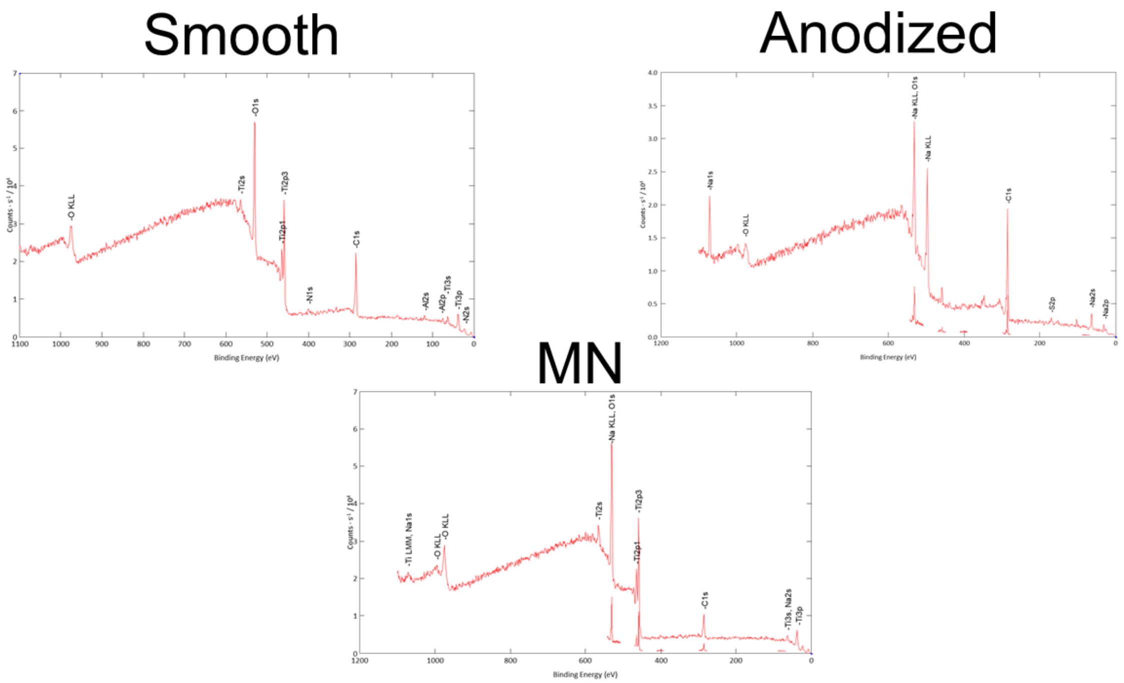

Supplementary Figure S1. Representative XPS Spectra of the three different surfaces used throughout the studies.

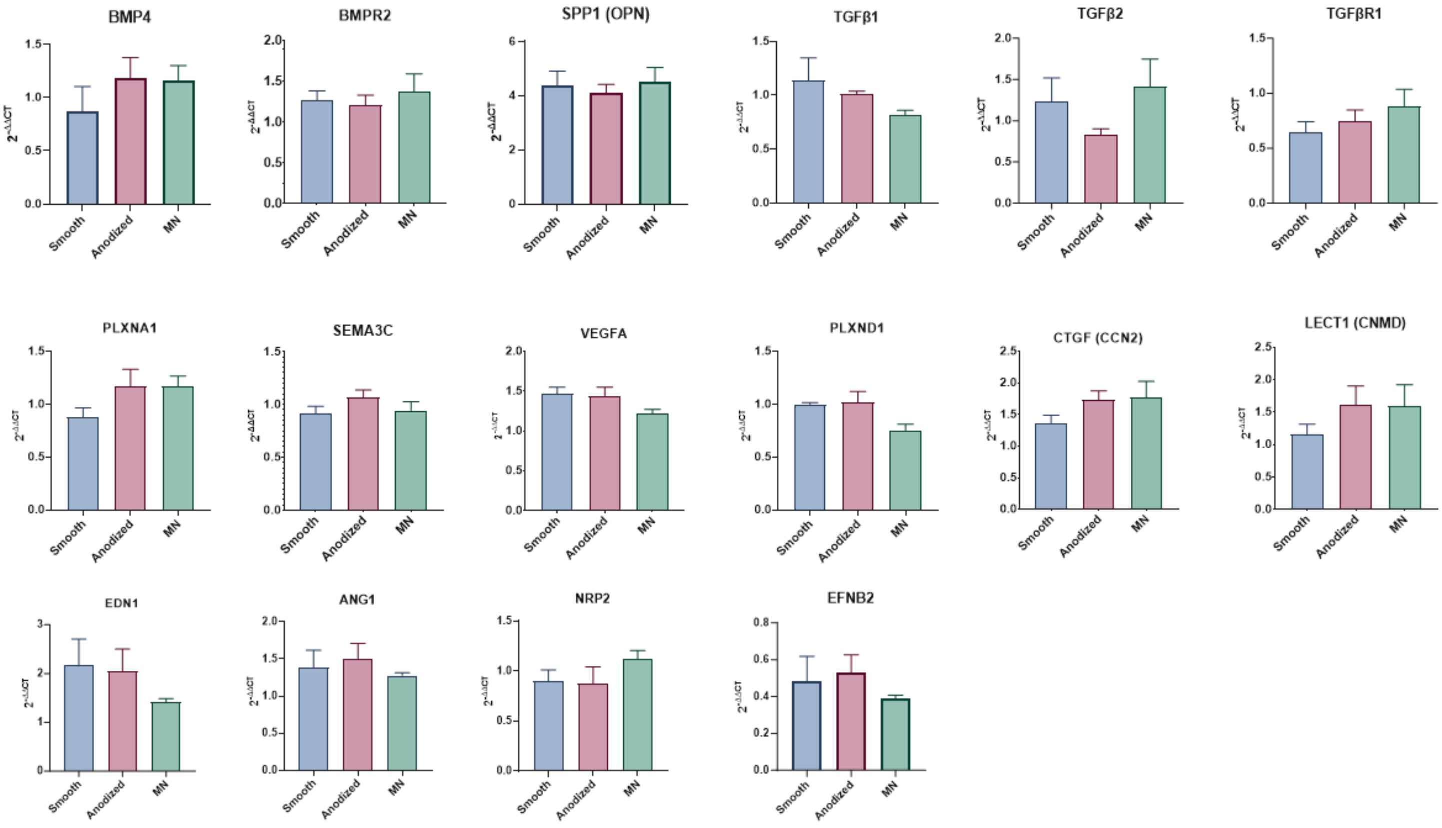

Supplementary Figure S2. Relative expression of osteogenesis and angiogenesis-related genes in human BMSCs cultured for 14 days on TCPS, smooth, anodized, or micro/nano-rough (MN) surfaces by RT-qPCR. Fold changes to TCPS were normalized to GAPDH and relative expression determined using the $2^{-\Delta\Delta CT}$ method. Values presented are means ± SE of twelve independent cultures per surface, pooled in duplicate for n of 6 per group. The group were evaluated using analysis of variance (ANOVA) with Tukey post-hoc test. Statistical significance was established at p-values equal to or less than 0.05 (*$p < 0.05$; **$p < 0.01$; ***$p < 0.001$; ****$p < 0.0001$).

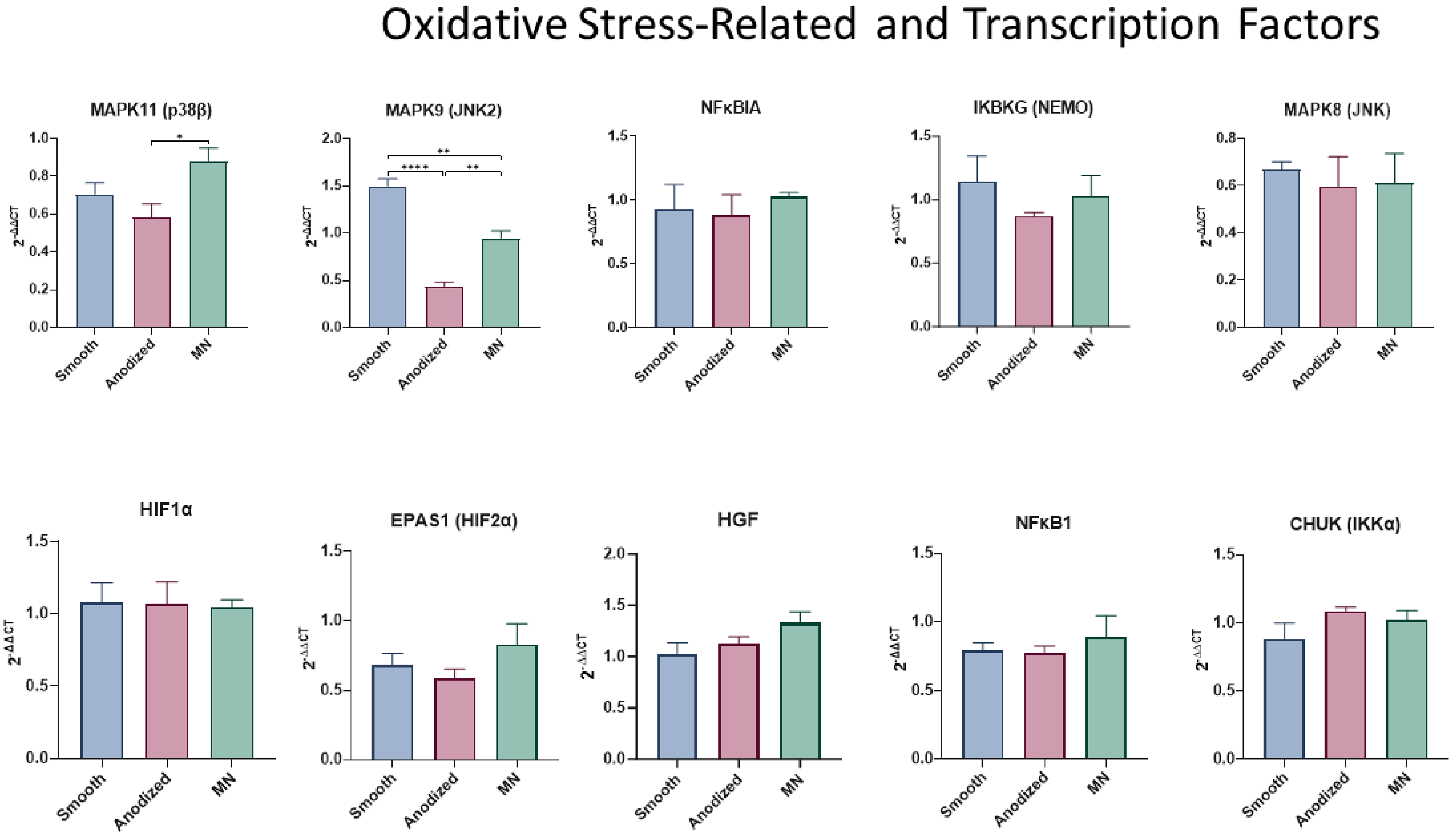

Supplementary Figure S3. Relative expression of oxidative stress-related and transcription factors and related genes in human BMSCs cultured for 14 days on TCPS, smooth, anodized, or micro/nano-rough (MN) surfaces by RT-qPCR**.** Fold changes to TCPS were normalized to GAPDH and relative expression determined using the $2^{-\Delta\Delta CT}$ method. Values presented are means ± SE of twelve independent cultures per surface, pooled in duplicate for n of 6 per group. Comparisons were made between test surfaces by one-way ANOVA with Tukey's post-hoc test (α=0.05).

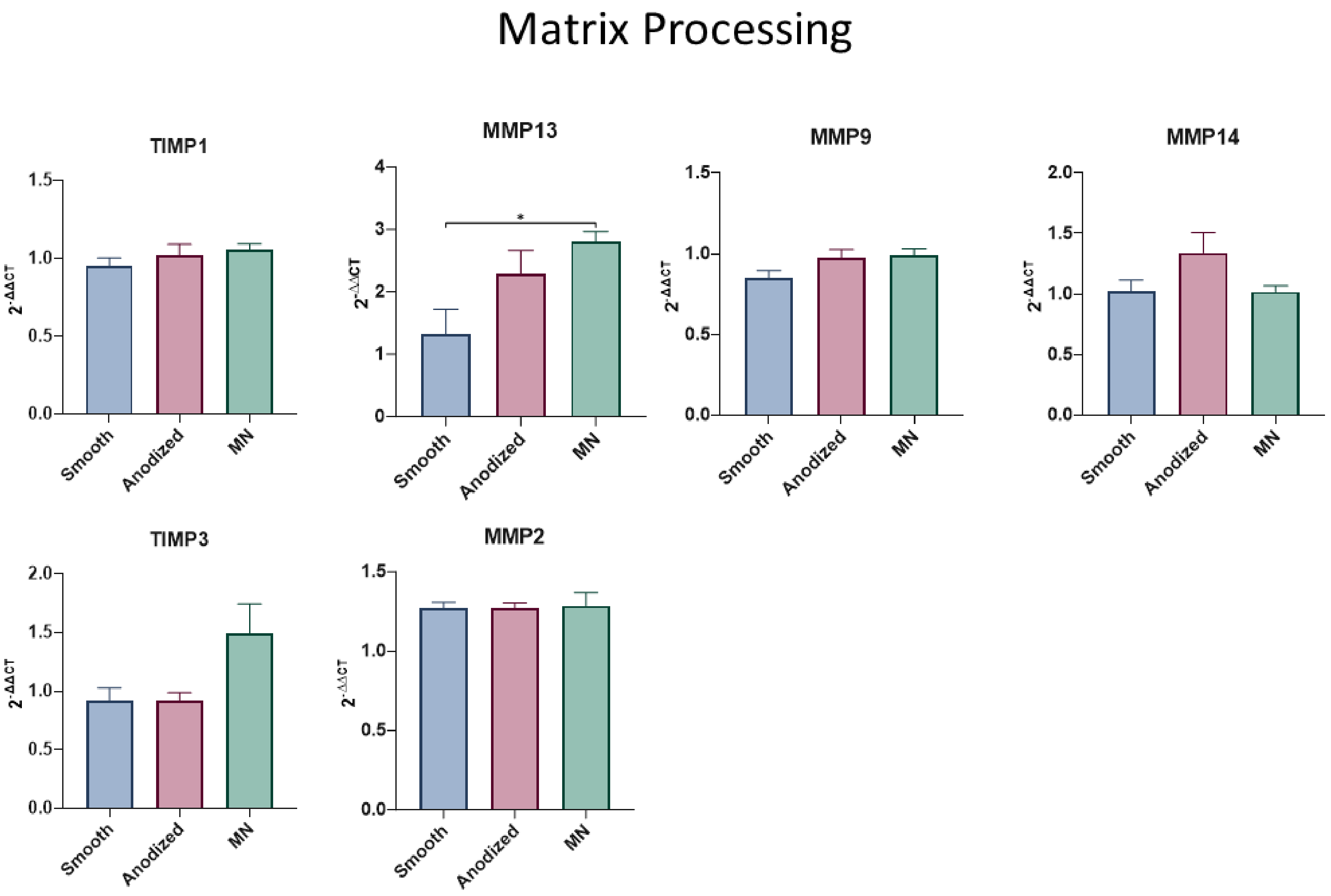

Supplementary Figure S4. Relative expression of extracellular matrix processing genes in human BMSCs cultured for 14 days on TCPS, smooth, anodized, or micro/nano-rough (MN) surfaces by RT-qPCR**.** Fold changes to TCPS were normalized to GAPDH and relative expression determined using the $2^{-\Delta\Delta CT}$ method. Values presented are means ± SE of twelve independent cultures per surface, pooled in duplicate for n of 6 per group. Comparisons were made between test surfaces by one-way ANOVA with Tukey's post-hoc test (α=0.05).

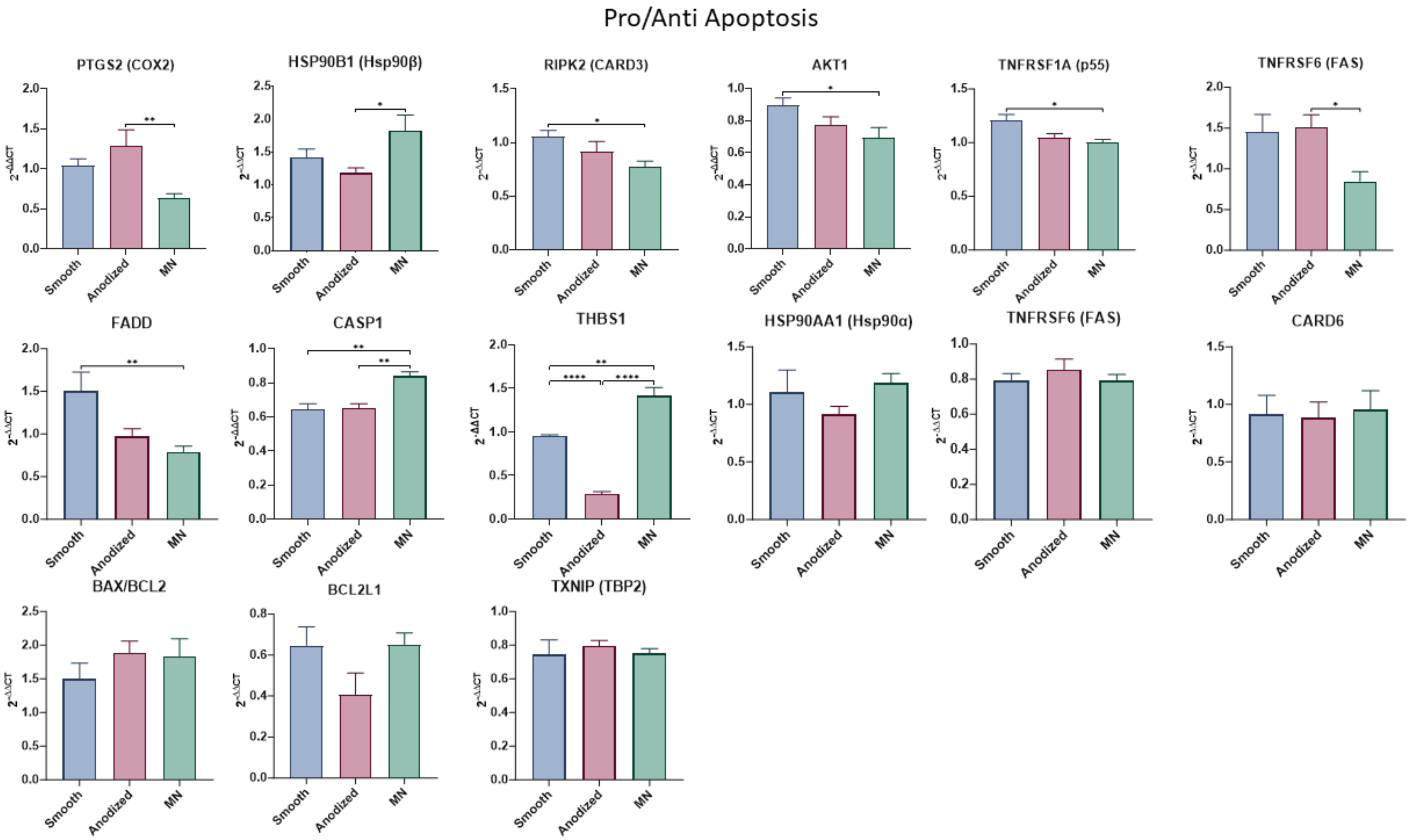

Supplementary Figure S5. Relative expression of pro and anti-apoptosis related genes in human BMSCs cultured for 14 days on TCPS, smooth, anodized, or micro/nano-rough (MN) surfaces by RT-qPCR**.** Fold changes to TCPS were normalized to GAPDH and relative expression determined using the $2^{-\Delta\Delta CT}$ method. Values presented are means ± SE of twelve independent cultures per surface, pooled in duplicate for n of 6 per group. Comparisons were made between test surfaces by one-way ANOVA with Tukey's post-hoc test (α=0.05).

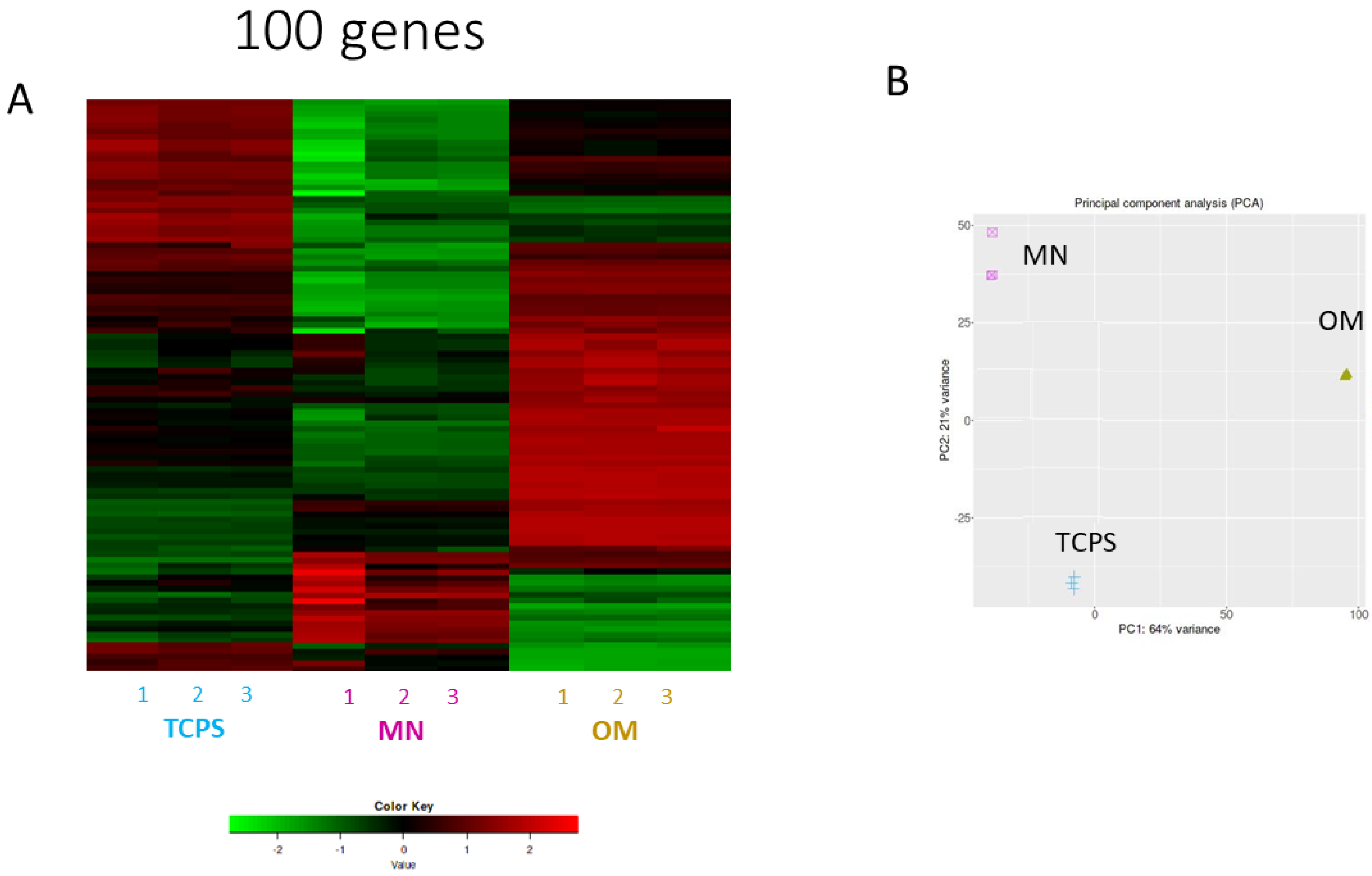

Supplementary Figure S6. Comparison of differentially expressed genes (DEGs) from a panel of 100 genes associated with osteogenesis. Human BMSCs were grown for 14 days on TCPS in osteogenic media (OM), on TCPS in growth media (TCPS) or on micro/nano-rough Ti6Al4V surfaces (MN) in growth media. (A) Clustering heat map and (B) principal component analysis (PCA) plot of expressed genes as determined by RNA-seq. Red and green in clustering heat map denote upregulated and downregulated genes, respectively, while grey denotes no significant difference between groups. Data in the PCA plot are from two independent experiments.
